# Biomarkers of Brain Injury: A Window on Mechanisms of Injury and Recovery in the Brain

**DOI:** 10.3390/brainsci12030362

**Published:** 2022-03-09

**Authors:** Sergio Bagnato

**Affiliations:** Unit of Neurophysiology and Unit for Severe Acquired Brain Injuries, Rehabilitation Department, Giuseppe Giglio Foundation, 90015 Cefalù, Italy; sergiobagnato@gmail.com

The decision-making process regarding management after severe acute brain injury is based on clinical evaluation and depends on the injury etiology as well as radiological and neurophysiological data [1,2]. Growing evidence suggests that several cerebrospinal fluid- and blood-derived biomarkers will be routinely used in the assessment of patients with acute brain injuries in the future. Compared with neuroimaging and neurophysiological data, biomarker data offer a very different perspective on brain injuries, focusing on sites and mechanisms of cellular damage. For example, among the most commonly examined biomarkers, neuron-specific enolase and ubiquitin carboxy-terminal hydrolase L1 are released with injuries affecting the neuronal cell bodies, whereas the neurofilament light chain (NFL) and tau proteins reflect axonal injury at different levels, and S100 calcium-binding protein B and glial fibrillary acidic protein are released with astroglial injury.

The examination of the specific biomarkers of brain injury is not limited to adult patients. In this Special Issue of *Brain Sciences*, entitled “Frontiers in biomarkers of brain injury,” Metallinou et al. [3] report on the use of serum activin A levels during the first 3 days of life in 96 preterm (<34 gestational weeks) infants, in order to identify infants who will develop neonatal brain injuries. Common brain injuries in preterm infants are intraventricular hemorrhage and periventricular leukomalacia. Intraventricular hemorrhage originates from the germinal matrix, a highly vascularized and transient structure in which neuronal and glial precursors are located before they migrate to other parts of the brain [4]. Periventricular leukomalacia derives from the high vulnerability of the white matter in preterm infants to hypoxic and ischemic damage [5]. Activin A is a member of the transforming growth factor-β superfamily; it promotes neuronal growth and differentiation in the developing brain and is upregulated in perinatal brain injury [6]. Metallinou et al. [3] showed that serum activin A levels were significantly higher in the first and second days of life in 29 preterm infants who later developed intraventricular hemorrhage or periventricular leukomalacia. These data identify activin A as a possible biomarker of neonatal brain injury in preterm infants.

Moreover, growing evidence suggests that acute brain injury is followed by chronic neurodegeneration [7]. Accordingly, biomarkers may also be applied in the evaluation of pathophysiological mechanisms operating in chronic brain injuries. In this Special Issue of *Brain Sciences*, Bagnato et al. [8] report on the assessment of serum NFL levels in 70 patients with prolonged disorders of consciousness following traumatic or hypoxic-ischemic brain injury. Compared with those of healthy matched controls, serum NFL levels were higher in these patients up to 6 months after injury. Moreover, NFL levels varied among patient subgroups with different injury severities and etiologies. These data suggest that severe traumatic and hypoxic-ischemic brain injuries trigger neurodegeneration with prolonged axonal injury. This neurodegeneration may counteract plastic changes required to recover from a disorder of consciousness [9], and changes in NFL levels could be used as a biomarker of injury and to evaluate the effectiveness of new neuroprotective strategies.

Biomarkers may also play a role in the evaluation of cognitive impairments after the occurrence of brain injury. Specifically, traumatic brain injury triggers neuroinflammation, which may affect patients’ long-term outcomes [10]. Malik et al. [11] performed a systematic review to identify changes in neuroinflammatory cytokines levels in patients with psychological symptoms after mild traumatic brain injury. They found that the upregulation of specific cytokines (interleukin-6, tumor necrosis factor-α, interleukin-10, C-reactive protein, and interleukin-1β) is associated with worse psychological outcomes, including increased risks of post-traumatic stress disorder and depressive symptoms, in patients with chronic mild traumatic brain injuries.

These examples from the papers published in this Special Issue of *Brain Sciences* highlight the breadth of the fields in which biomarkers have potential applications regarding brain injury, ranging from infants to adult patients and from traumatic to hypoxic-ischemic and acute to chronic injuries. There are several factors that still limit the routine use of several biomarkers in clinical practice, including high costs and the need for specialized skills and specific equipment. It is expected that these obstacles will be overcome in the next few years for several biomarkers, allowing their introduction into routine clinical practice.
